# Innovative DendrisChips^®^ Technology for a Syndromic Approach of In Vitro Diagnosis: Application to the Respiratory Infectious Diseases

**DOI:** 10.3390/diagnostics8040077

**Published:** 2018-11-11

**Authors:** Alice Senescau, Tatiana Kempowsky, Elodie Bernard, Sylvain Messier, Philippe Besse, Richard Fabre, Jean Marie François

**Affiliations:** 1 DENDRIS SAS, F-31670 Labège, France; asenescau@dendris.fr (A.S.); tkempowsky@dendris.fr (T.K.); ebernard@dendris.fr (E.B.); messier_sylvain@yahoo.fr (S.M.); 2 Département Génie Mathématiques et Modélisation, Fédérale Université of Toulouse, F-31077 Toulouse, France; philippe.besse@insa-toulouse.fr; 3 BIOPOLE, 2, F-31670 Labège, France; richard.fabre@biopole.fr; 4 LISBP, Fédérale Université de Toulouse, CNRS, INRA, INSA, F-31077 Toulouse, France

**Keywords:** in vitro diagnostic (IVD), syndromic approach, DNA, dendrimer, PCR, biochips, machine-learning methods

## Abstract

Clinical microbiology is experiencing the emergence of the syndromic approach of diagnosis. This paradigm shift will require innovative technologies to detect rapidly, and in a single sample, multiple pathogens associated with an infectious disease. Here, we report on a multiplex technology based on DNA-microarray that allows detecting and discriminating 11 bacteria implicated in respiratory tract infection. The process requires a PCR amplification of bacterial 16S rDNA, a 30 min hybridization step on species-specific oligoprobes covalently linked on dendrimers coated glass slides (DendriChips^®^) and a reading of the slides by a dedicated laser scanner. A diagnostic result is delivered in about 4 h as a predictive value of presence/absence of pathogens using a decision algorithm based on machine-learning method, which was constructed from hybridization profiles of known bacterial and clinical isolated samples and which can be regularly enriched with hybridization profiles from clinical samples. We demonstrated that our technology converged in more than 95% of cases with the microbiological culture for bacteria detection and identification.

## 1. Introduction

Infectious diseases are associated with multiple pathogens that can be bacteria and viruses [1,2]. In clinical laboratories, the current practice for their identification relies on microbiological or biochemical techniques including microbial cultures, microscopy, antigen detection, serology and more recently mass spectrophotometry. However, these methods only identify cultivable pathogens in a low throughput manner (i.e., one microbe per culture condition), which can be ineffective when the patient has been subjected to an antimicrobial therapy prior to the culture test. In addition, the turnaround time to deliver the diagnostic is rarely achieved within the required time for patient management. To circumvent these limitations, rapid and affordable technologies able to identify and discriminate in a single test several potential pathogens are strongly welcomed by the medical and clinical profession [3]. According to Infectious Diseases Society of America and the US Food and Drug Administration (FDA) [4], and recently reviewed by Raman et al. and Gray et al. [5,6], the syndrome-based diagnosis is foreseen as the new paradigm for in vitro diagnostic in clinical microbiology. This new approach implies molecular multiplexing detection techniques that will encompass nucleic acid extraction, liquid handling, molecular amplification and identification. Furthermore, these molecular techniques should be amenable to partial or complete automation and high throughput assays.

Multiplexing endpoint PCR technologies are nowadays the most advanced technology for molecular in vitro diagnostic (IVD) and some of them have already been approved by FDA [3,6]. Among others, we can cite the multiplex real-time PCR Respi Finder^®^ SMART 22 from Pathofinder, (The Netherlands), the Seeplex^®^ RV15 one step ACE detection from Seegene (South Korea), the FilmArray^®^ RP (Respiratory Panel) from BioFire Diagnostics (USA), the GeneXpert^®^ IV from Cepheid (USA), and the QIAstat-Dx system from Qiagen (Germany). Main advantages of these multiplex PCR technologies are easy to use, high sensitivity that facilitates detection of pathogens from minute amounts of DNA, and delivery of a result in matter of few hours. Many papers have been published on the comparison of these multiplex PCR technologies with respect to their reliability, sensitivity and detection limit. They notably highlight their rapidity and accuracy over standard methods based on microbiological cultures [7,8,9,10,11,12]. However, a main drawback of these technologies is their relatively low throughput in terms of number of pathogens to identify at once, together with the prohibitive cost of these technologies. In addition, some multiplex PCR techniques such as those proposed by Biofire, Cepheid or Qiagen are fully integrated which does not allow cross-checking the same sample with other independent methods. In addition, the integration aspects of these technologies make them relatively expensive and only accessible to large hospitals or clinical laboratories.

An alternative method that can overcome some of the drawbacks listed above is the DNA microarrays technology in which tens to thousands of probes (short sequence of oligonucleotides) immobilized on a solid support are hybridized with their corresponding nucleic acid targets (DNA/RNA) [13]. This technology developed more than 25 years ago by Affymetrix [14] has been largely exploited in academic research and is still subjected to several technical developments, including microstamping, nano-tip printing, electro-printing, etc. [15,16]. However, application of microarray technology in medicine, environment or food security is almost non-existent, likely because this technology is apparently complex, not robust and not user-friendly yet [17]. Few companies have been engaged in this technology. An interesting, yet expensive technology, is proposed by GenMark diagnostic Inc. (USA). The principle of their technology resides in the electrical capture of the hybridization signal between viral DNA/RNA targets and the ferrocene-labeled probes that are immobilized on a gold electrode surface. This biochip prototype is embedded in an eSensor^®^ XT-8 cartridge which constitutes the base module of the e-Sensor XT-8 instrument. The high throughput of this system is provided by the capacity to handle up to 24 cartridges at once, with each cartridge being able to detect up to 25 different targets including bacteria and virus [18,19]. The xTAG RVP is another DNA microarray technology developed by Luminex Corporation (USA). This technology is a bead array-based system divided into two main junctures: a multiplex RT-PCR that generates target specific amplicons (TSE), followed by target specific primer extension reaction to create biotinylated extension products flanked with a specific DNA Tag-sequence. The extension products are combined with anti-tag conjugated beads array to allow specific tag/anti-tag hybridization. The beads are sorted out and detected with a fluorescent reporter molecule on the Luminex^®^ xMAP flow cytometer [20,21,22]. While these commercially available systems have their unique technological advantages, such as high automatization, faster deliverance of the diagnosis results (i.e., in the range of 1 to 6 h according to the technology) and high throughput samples analysis per day when compared to conventional cultures methods [3,5], they have in common being very expensive which restricts their use in large hospitals. Besides, most of these multiplex molecular techniques are focused on the identification of viruses in respiratory infection, likely because clinical symptoms of pneumonia for instance can be associated with bacteria or viruses [23], and hence a rapid diagnostic of the presence/absence of virus could avoid misuse of antibiotic therapy which is the main cause of the emergence of resistant bacteria.

Given the fact that syndromic testing is becoming increasingly attractive in the field of clinical microbiology, there is still room to innovate in multiplex molecular detection and to propose alternative, cheap and user-friendly, technology for rapid in vitro diagnosis. In this report, we describe a prototype system that has been developed for syndrome-based detection of bacterial species potentially associated with the respiratory tract infection. We focused the development and validation of our technology on this infectious disease because it is the leading cause of death in developing countries causing around 4 million deaths annually [2,24]. The technical originality of our prototype system stands in our proprietary technology employing the dendrimer chemistry that significantly enhances the sensitivity of the detection [25]. Our technology also relies on the simplification of PCR step, which is performed on genetic variable regions in the ribosomal DNA (16S rDNA) of bacterial genome using only a couple of primers. The third originality resides on the application of statistical learning methods as decision making support to the clinician by providing a probability result of presence/absence of bacterial pathogens. Overall, the whole process starting from DNA extraction takes less than 4 h. We have applied our DendrisChip^®^ technology to detect and discriminate pathogen bacteria in clinical specimens suffering from respiratory tract infections. Comparison of our technology with classical microbiological methods showed a convergence of more than 95% of results between the two methods. Our methodology is also discussed in terms of easy-to-use and robustness.

## 2. Materials and Methods

### 2.1. Bacterial Strains and Clinical Specimens

The bacterial strains listed in Table 1 and Table 2 were used in this study. They were obtained as pure culture from clinical laboratories of Toulouse. The 16S rDNA regions targeted for designing the oligonucleotide probes (see below) have been sequenced from these pure clinical cultures. A total of 238 samples from patients collected by sputum, tracheal or bronchial aspirate, bronchoalveolar lavage (BAL) or ENT (Ear-Nose-Throat) swabs were provided from independent clinical laboratories of Toulouse. They were analyzed simultaneously by conventional bacteriological cultures. This study had thus a double-blind design in which the laboratory biologist performing the routine analysis was unaware of the microarray analysis carried out at Dendris SAS, and reciprocally.

### 2.2. Species-Specific and QC Probes Design

A total of 169 probes were designed on hypervariable region of the 16S rDNA from the inclusion bacterial strains listed in Table 1. The hypervariable regions of the 16S rDNA have been sequenced prior to make probes design. Multiple alignment analysis using ClustalW was applied to the probe design. The uniqueness of the sequence of each probe was queried against sequences in Genbank database with a BLAST search. Probe quality criteria, namely length of the oligonucleotide between 20 and 25 nucleotides long, equal melting temperature, lack of hairpin and dimer formation, were assessed with Primer 3plus [26,27]. Two types of artificial probes were further designed for quality control of the process. The probes were purchased from Eurofins (Les Ulis, France) with their 5′ end NH2-modified.

### 2.3. Manufacture and Format Design of the DendrisChip^®^

**Figure 1:**
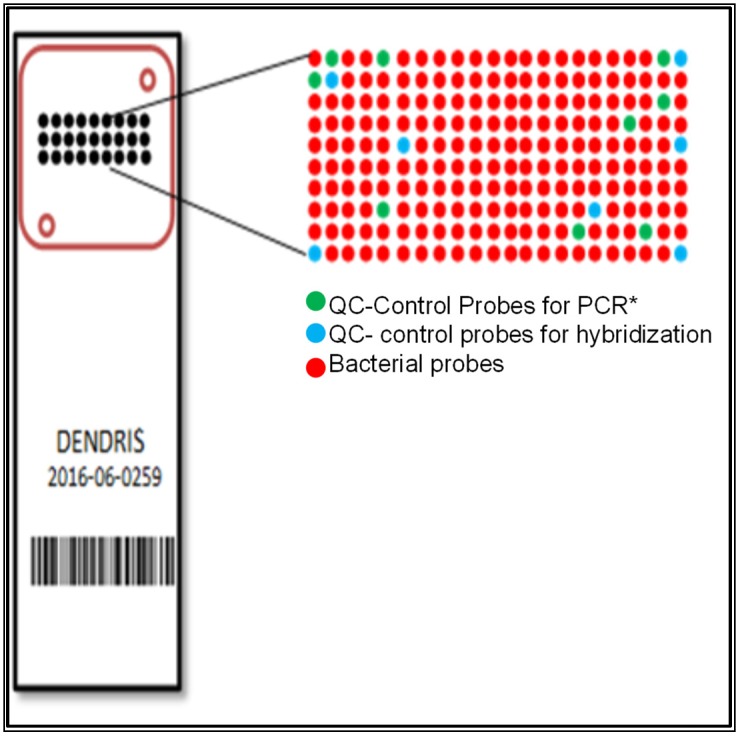
Description of the DendrisChips^®^; QC mean quality control.

Manufacture of the DendrisChips^®^ was carried out essentially as described by Trevisiol et al. [28]. Briefly, the probes were dissolved at a concentration of 50 µM in 0.15 M phosphate buffer pH 9.0 and printed in triplicate randomly on dendrislides by piezo electrical dispensing with the SciFlexArrayer SX robot from Scienion (Berlin, Germany). The average size of the spots was 150 ± 10 µm and each spot was spaced by 500 µm. The capture probes were arranged as schematically described in Figure 1. The seven blue spots were a 25-mer synthetic probe (CIH_ol) which was spotted for correct positioning of the DendrisChip^®^ during the image processing. This synthetic probe is a reference for validation of the hybridization step by recognition of a complementary oligonucleotide added to the hybridization buffer (see Figure 1). The green spots corresponded to the QC probe used for internal control to evaluate the suitability of the PCR amplification carried out on the extracted DNA samples. This QC probe of 25-mer length was designed to hybridize with a 246 bp synthetic DNA fragment, amplified with a specific pair of primers that is present in the PCR reaction mixture.

### 2.4. DNA Extraction and PCR Amplification

DNA was extracted using DNeasy Blood & Tissue Kit from Qiagen (Courtaboeuf, France) according to the manufacturer’s instructions for bacterial DNA extraction and in the presence of proteinase K which has been described as important to extract DNA from gram-positive bacteria [29]. The DNA from reference strains was isolated from one colony taken from Luria broth (LB) agar plates mixed with 200 µL of DNase-free water. For DNA from clinical samples, this first step depended on the type of samples. When originated from swabs, they were submerged in 200 µL of PBS 1× and a strong shaking was performed to obtain a bacterial suspension for the next steps of extraction. Samples obtained by sputum, tracheal or bronchial aspirate and bronchoalveolar lavage were incubated 15 min at 95 °C with 200 µL of PBS 1×. The next steps of extraction were common to all types of sampling. After centrifugation for 10 min at 3000× *g*, the pellet was resuspended in 180 µL of an enzymatic lysis buffer (lysozyme to 40 mg/mL in 20 mM Tris-Cl, pH 8.0, 2 mM Sodium EDTA, 1.2% Triton^®^X-100) and incubated at 37 °C for 30 min. Then, the manufacturer’s protocol for bacterial DNA extraction was followed and the DNA was eluted in 200 µL. PCR amplification was made on the 16S rDNA gene using previously described primers 16S-27F [30] and 16S-1392R [31] with the reverse primer labeled at its 5′ end with Cy5. The PCR reaction was carried out in total volume of 50 µL in 1× PCR buffer (Sigma-Aldrich, Saint Quentin en Yvelines, France) containing 3.75 U of JumpStart Taq DNA Polymerase (Sigma-Aldrich), 3 mM MgCl2 (Sigma-Aldrich), 0.2 mM deoxynucleotides (dNTPs) (Sigma-Aldrich), and 10 pmol of each primer (Eurofins) and DNA (5–100 pg) extracted from samples. The amplification was done with a touch-down PCR in a GTQ-Cycler 96 from Bruker-Hain Diagnosis (Nehren, Germanny) using the following program. The first ten cycles started with a denaturation at 94 °C for 30 s, followed by a touch-down primer hybridization from 60 °C to 50 °C for 30 s with decreasing temperature of 1 °C per cycle and it ended with an elongation step at 72 °C for 81 s. Then, 25 cycles were performed under the following conditions: 30 s at 94 °C, 30 s at 50 °C and 81 s at 72 °C. The PCR amplicons were purified with MinElute PCR Purification Kit purchased from Qiagen (Courtaboeuf, France) according to manufacturer’s instructions. The DNA concentration and incorporation of Cy5 were determined by UV spectrometry (NanoDrop 2000 from ThermoScientific France, Villebon Sur Yvette, France).

### 2.5. Hybridization and Fluorescence Intensity Scanning

The purified PCR amplicons (10 µL) were heated for 2 min at 95 °C and added in 320 µL of hybridization buffer containing 5× Denhardt’s solution (1% Ficoll (type 400), 1% polyvinylpyroolidone and 1% Bovin Serum Albumin), 2.5 × SSC, 100 µg/mL salmon sperm DNA and the 5′ CY5-labeled oligonucleotide complementary of CIH_ol at a final concentration of 1 nM. This mixture was loaded on the area of DendrisChip bearing the probes and protected by a coverslip. Then, the chips were incubated at 60 °C and 250 rpm for 30 min (Thermo Shaker PHMP from Grant Instruments, Cambridge, UK). The slides were washed two times with 25 mL of the first washing buffer (2 × SSC, 0.2% SDS) for 3 min at RT and 300 rpm (Rotamax from Heidolph Instruments Schwabach, Germany), followed by one-time 25 mL of the second washing buffer (0.2 × SSC), for 3 min at RT and 300 rpm. The slides were then air-dried by centrifugation and scanned at 10 µm resolution and at 100% of photomultiplier tube gain using the confocal laser scanner DendriScan (Innopsys, Carbonne, France). Fluorescence intensities of spots scanned were numbered by the DendriSoft software version 1.0.0 (Innopsys, Carbonne, France), were converted into numerical values and then stored in .gpr data sheets. For statistical analysis, as described below, raw fluorescence data for each spot minus its surrounding fluorescence background were used.

### 2.6. Evaluation of the Limit of Detection

The limit of detection was tested using dilution from a stock solution of five bacteria from our panel. From a culture of each bacteria, a suspension in saline solution was carried out to achieve a turbidity equivalent which corresponds to 2 × 10^8^ CFU/mL according to McFarland range (CASFM, 2018). Serial dilutions were realized to obtained concentrations of 10^8^–10^2^ CFU/mL. DNA was extracted from serial culture dilutions and further processed according to the DendrisChip protocol described above. The fluorescence after hybridization were compared to the statistical model built by Random Forest to predict the bacterium detected. We considered as the limit of detection (LOD) the lowest concentration from which bacteria could be detected with a probability of >0.6.

### 2.7. Data Analysis and Pattern-Mapping Statistical Analysis

For discrimination analysis, a statistical model (profile) specific to each bacterium was constructed based on Random forest (RF) algorithm [32]. To this end, we used the caret e1071 and random forest packages within the R statistical analysis software [33] available at http://www.r-project.org. RF is a versatile machine learning method capable of performing both regression and classification tasks. It is an “ensemble” approach that generates many decision tree models and aggregates their results. Each of the classification trees is built using a bootstrap sample of the data, i.e., hybridization intensities from bacteria samples, and at each split the candidate set of variables (i.e., probes) is a random subset of the variables. Each tree is grown fully with no pruning. The algorithm yields an ensemble that can achieve both low bias and low variance. The prediction error is computed using data that are not in the bootstrap sample (out-of-bag (OOB)). RF also measures variables (probes) importance that allows selecting the most discriminant ones for each bacteria profile. We compared the two proposed methods to calculate variable importance, namely GINI index of node impurity and the prediction error by permutation of OOB data, and found the latter as the most relevant for this application.

## 3. Results

### 3.1. Validation of the Bacterial SPECIES-specific Oligonucleotide Probes on the DendriChip^®^

The use of 16S rDNA to identify bacteria species has several advantages such as internal transcribed spacer hypervariable regions, abundance of nucleic acid sequences of bacterial genomic regions in databases and availability of reported specific capture probes and universal primers. Nonetheless, several studies on 16S rDNA-based detection have shown inevitable non-specific binding because sequences in this region do not show enough sequence diversity which can hamper the species-specific bacteria detection [34,35]. Another and too often forgotten problem when dealing with hybridization between complementary DNA strands is the importance of the surface chemistry onto which nucleic acid fragments termed oligoprobes are immobilized. Indeed, several reports have shown that the chemistry used to retain the nucleic acids on the surface can dramatically affect the specificity and selectivity of the probes with their complementary DNA/RNA targets [36,37,38]. Taking into account these problems, we designed a large set of 25 mer probes from sequenced 16S rDNA region of eleven pathogen bacteria (inclusion bacteria) commonly found in respiratory tract infection with Primer3Plus software (Table 1). We retained a set of 169 probes whose specificity for these 11 bacteria were further queried against sequences from GenBank databases NCBI and EMBL-EBI.

The first step for the design of the DendrisChips^®^ was to retain the probes that were the most specific to each bacterium. Technically, we printed several Dendrislides with the 169 probes and hybridized CY5-labeled PCR amplicons from DNA extracted from our “inclusion” bacteria (Table 1). To validate the better accessibility of the probes to the DNA targets with DendrisChips^®^, we also spotted these probes on commercially available chemically-activated glass slides. We clearly found that the hybridization signals of a *Legionella pneumophila* PCR amplified DNA fragment on either aminosilane or epoxy-coated slides were much lower than with the DendrisChips^®^ (see Figure S1 in the Supplementary Materials). After this first stage, we excluded 101 probes due to low or absence of fluorescent signals with some of the designed probes on the targeted bacteria. We then evaluated the species-specificity of the remaining 68 probes on the inclusion panel of bacteria by carrying out hybridization assays using PCR amplicons from “an exclusion panel” of bacteria (Table 2). These latter are bacteria that belong to the same genus but are different species such as *Klebsiella pneumoniae* and *Klebsiella oxytoca*, and *Staphylococcus aureus* and *Staphylococcus epidermis*. With respect to the bacterial species reported in Table 2, we did not find any cross-hybridization between the inclusion and exclusion bacteria (data not shown).

### 3.2. Limit of Detection, Quality Control and Robustness of the DendrisChips^®^

We evaluated the detection limit of our microarrays system using DNA extracted from pure bacterial colonies that was diluted from 12.5 to 0.0125 ng per mL before performing the PCR. By fixing a positive hybridization signal at three times the background, a limit of detection (LOD) in the range of 0.2 pg DNA per reaction was recorded for all targeted bacterial strains, with the exception of *Staphylococcus aureus* (Sau) which required about 5–10 times more DNA. Taking into account that one bacterial cell contains around 5 fg (depending of the genome size, but as a rule of thumb, 1 Mbp DNA corresponds to 1 fg), the LOD of the Dendrischip could be estimated in the range of 10^2^–10^3^ CFU/mL. However, to approach the clinical samples condition in which bacteria can be present in a range from <10^2^ to >10^6^ CFU/mL, we actually evaluated the detection limit by extracting DNA content from culture containing bacteria in this CFU range. As reported in Table 3, the LOD was one log above that obtained from direct serial dilution of DNA and was about one log higher than that obtained using a real-time PCR.

A second important validation was to determine the robustness of our DendrisChip^®^ in terms of repeatability and reproducibility of the results. For the reproducibility test, DNA of eight different bacteria was extracted and this was repeated with three independent colonies from these eight bacteria. After PCR amplification, labeled DNA fragments were hybridized on three independent DendrisChip^®^. Correlation coefficients (*r*^2^) between slides taken two by two for each of the eight bacteria were all above 0.8 (see Figure S2, in the Supplementary Materials). The repeatability was evaluated by carrying out the complete procedure using the same DNA from six different bacteria, but in this case, PCR amplification and hybridization were performed at Day 0, 3 and 10, keeping the extracted DNA at −20 °C between each interval of time. Again, a correlation coefficient between these independent experiments was above 0.8 (see Figure S3, in the Supplementary Materials).

To assess the robustness of our DendrisChip technology, we introduced two quality controls (QC) in the process. A “hybridization” QC was implemented by spotting a 25-mer QC probe that is complementary to the CY5-labeled CIH_ol target at each corner of the spotting area. A second QC was set up to assess the PCR amplification step on the DNA extracted from biological samples. To this end, a 25 “mer” probe that hybridizes with a 246 bp synthetic gene was spotted randomly on the Dendrislide (Figure 1). The amount of synthetic gene added to the biological samples was determined experimentally to be 8 pg DNA such that the fluorescent signal obtained after PCR amplification was in the range of those obtained after amplification of bacteria DNA that could be present in biological samples.

### 3.3. Machine-Learning Method to Identify Discriminant Probes for Bacterial Species

**Figure 2:**
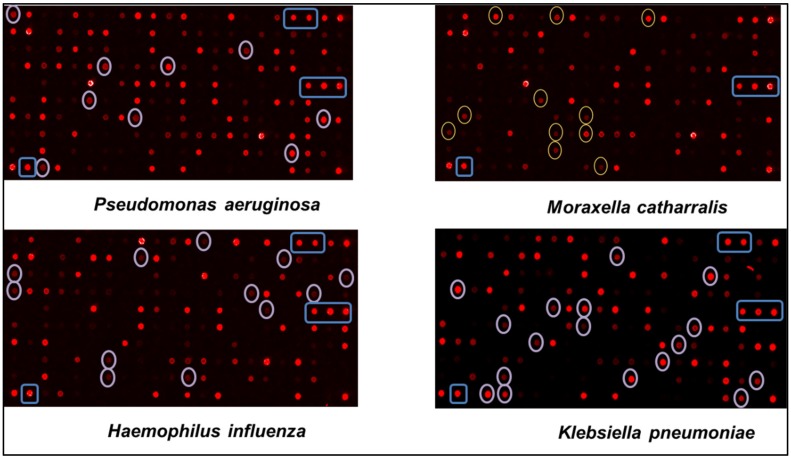
Example of assignment of specific oligonucleotides probes for four bacterial strains. Each picture shows the results of hybridization from a single bacterial strain taken from a pure culture. The probes specific for the bacteria are circled. The blue boxes indicate unspecific fluorescent signals.

The fact that species-specific probes were found for each of the eleven pathogen bacteria was already a good achievement (Figure 2). However, we often found probes designed originally for a given species that showed fluorescent signals to many bacteria (see rectangle box in Figure 2).

**Figure 3:**
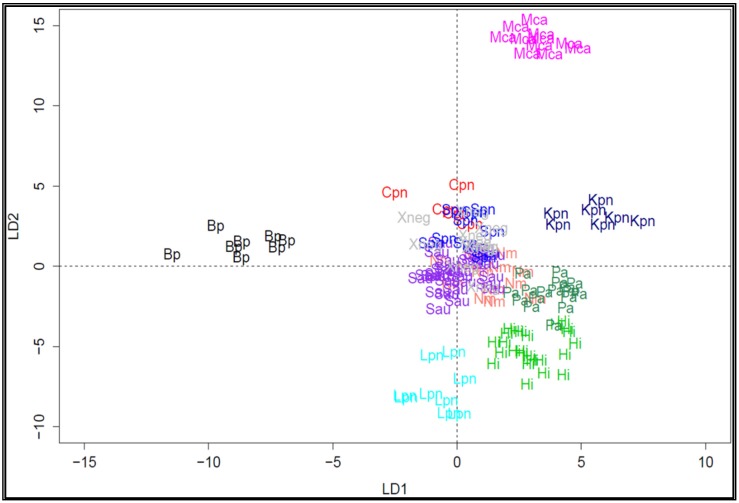
Two-dimensional projection of the hybridization profiles for 166 samples using canonical discriminant analysis. The samples that have been used to generate the training set are a combination of pure bacterial strains and clinical specimens in which bacterial species have been also identified by microbiological method. Bp, *Bordetella pertussis*; Cpn, *Chlamydiae pneumoniae*; Hi, *Haemophilus influenzae*; Lpn, *Legionella pneumophila*; Kpn, *Klebsiella pneumoniae*; Mca, *Moraxella catharralis*; Spn, *Streptococcus pneumoniae*; Nm, *Neisseria meningitidis*; Sau, *Staphylococcus aureus*; Pa, *Pseudomonas aeruginosa*.

Therefore, to assign a specific signature for each bacterium, we built a database that initially contained 166 hybridization patterns and which was obtained from pure culture bacteria strains, known bacterial strains spiked in DNA extracted from respiratory tract of healthy person and clinical samples from which the bacterial species had been rigorously identified and confirmed by standard microbiology culture or by PCR. As shown in Figure 3, a two dimensional projection of the dataset using canonical discriminant analysis [39] allowed discriminating some but not all pathogens. Discrimination was noticed with *Bordetella pertussis* (Bp), *Haemophilus influenzae* (Hi), *Legionnella pneumophila* (Lpn), *Klebsiella pneumoniae* (Kpn), and *Moraxella catharralis* (Mca) but the other six bacteria were found in the same cluster, which precludes using this statistic linear method for complete discrimination.

We therefore employed Random Forests (RF) method [32] since this machine (statistical) learning method is more dedicated to prediction problems that are non-linear and that involve complex high-order interaction effects, which is actually the case with microarrays [40]. With this method, we generated training datasets employing the hybridization data from a 10-fold cross-validation experiment using inclusion and exclusion bacteria. The training set from this cross-validation was equally divided into 10 different data subsets. Nine out of ten were used to train the classifier, while the tenth subset was used as the test set. This procedure was repeated ten times, with a different subset being used as the test set. According to this process, a two-class (present/absent) random forest model was built for each bacterium with t decision trees, generated at random, using a different subset of the training dataset with replacement to train each tree. The remaining training data (Out-Of-Bag (OOB)) were used to estimate prediction error and variable (i.e., probe) importance for each bacterium. At each node of a tree, a number m of variables was selected at random, where m < M (total number of variables). The node was split using the best variable among the m ones. Each tree was grown to the largest extent possible. Thus, when a new sample entered the prediction system, the corresponding hybridization data of this sample run down all of the t trees to yield a result that is obtained by aggregating the predictions of the t trees (i.e., majority votes for classification). This process led to a probability of the presence or absence of any of the 11 bacteria in the sample. With this reference dataset, the random forest (RF) model showed an average misclassification rate of less than 10% for eight of the 11 bacterial strains. In the case of *Legionella pneumophila*, *Chlamydiae pneumoniae* and *Streptococcus pneumoniae*, this rate reached 20%, which could be explained by a reduced signal intensity of the probe sets due to matrix effects and/or a low number of independent samples in the training dataset.

**Figure 4:**
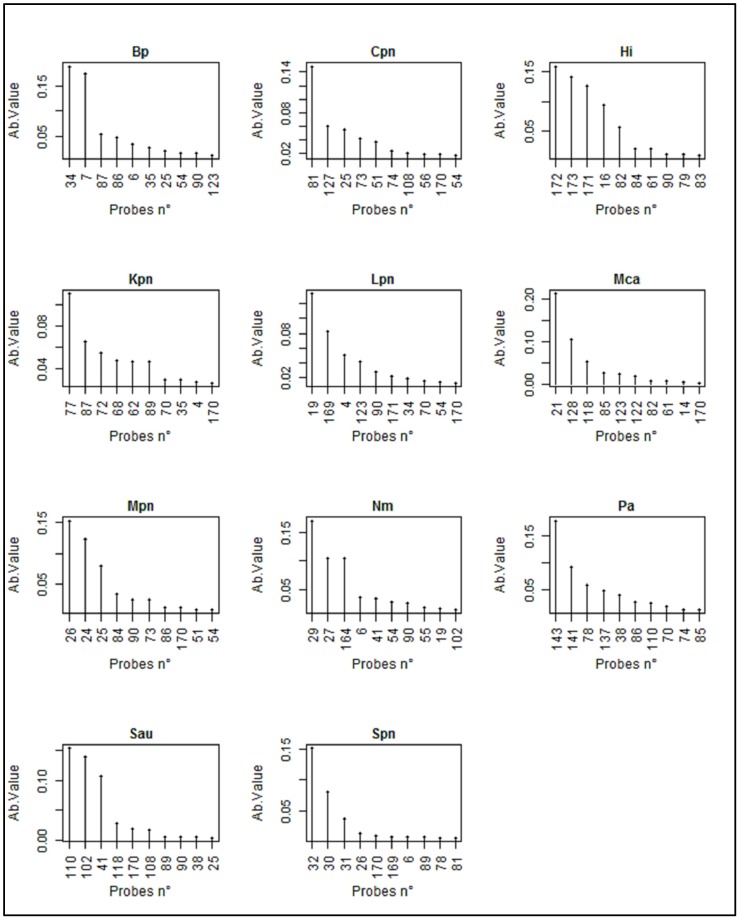
Probes patterns for the 11 pathogen bacteria obtained by random forests statistical analysis. The probes are ordinated in the X-axis by their importance value (Y-axis arbitrary unit) determined by the RF model as described in Materials and Methods. Bp, *Bordetella pertussis*; Cpn, *Chlamydiae pneumoniae*; Hi, *Haemophilus influenzae*; Kpn, *Klebsiella pneumoniae*; Lpn, *Legionella pneumophila*; Mca, *Moraxella catharralis*; Mpn, *Mycoplasma pneumoniae*; Nm, *Neisseria meningitidis*; Pa, *Pseudomonas aeruginosa*; Sau, *Staphylococcus aureus*; Spn, *Streptococcus pneumoniae*.

A second objective of using RF model is to be able to obtain for each bacteria a specific signature that allows discriminating each of them. To this end, we employed the measure of “variable (i.e., probe) importance” which was calculated from hybridization profiles as follows. For each tree in the forest (i.e., hybridization profiles of the bacterium), the out-of-bag prediction error (OOBoriginal) was recorded. Then, a random permutation of the values for the j^th^ probe was generated and the out-of-bag prediction error (OOBpermuted) was computed again with this perturbed dataset. This permutation disturbed the model and therefore increased the prediction error. The importance score for the jth probe was computed by averaging the difference in the out-of-bag error before and after the permutation over all trees and normalized by the standard deviation of the differences. Thus, the more the prediction is degraded (bigger OOBpermuted error), the more important the jth variable is for the discrimination. This RF model led us to generate for each bacterial strain an assignment of max 10 probes (Figure 4). Taking into account that high specificity requires an arbitrary variable ≥0.10, one can see that 8 out of the 11 selected bacteria were fingerprinted by two, three or even four probes. Exception was found with *C. pneumoniae* (Cpn), *K. pneumophila* (Kpn) and *L. pneumophila* (Lpn) for which one probe had a very high arbitrary value, indicating a high species-specificity. Nonetheless, the 2–3 other probes that had a value in the range of 0.05 were found to be useful in the assignment and discrimination of these bacteria.

### 3.4. Identification of Bacterial Species in Clinical Samples Based on Random Forests Model

We then evaluated our technology on true clinical samples obtained from six different laboratories in the Toulouse area. A total of 238 anonymous clinical samples were provided by these laboratories. These samples were distributed into 43 bronchoalveolar lavage (BAL) obtained from frozen-specimen collection, 43 were collected as sputum, 66 as throat swabs, 33 as nasopharyngeal swabs, 20 as wound swab, 15 as ear swabs, 5 as mouth swabs, 5 as tongue swabs, 1 as eye swab, 1 as tracheal aspirate, 6 as bronchial aspirates samples and finally 20 were “negative” samples (i.e., obtained from consenting healthy persons). In parallel and/or prior to being processed by our technology, these samples were analyzed by the provider laboratory using their standard microbiological cultures. Overall, our technology revealed the presence of one pathogen in 49% of the 238 specimens and two pathogens in 7% of this total sampling. In comparison, the microbiological result given by the laboratories indicated the presence of at least one pathogen in 53% of the 238 samples. Thus, at this point, the convergence between the two methods could be estimated to 97%. However, this high convergence value does not inform us about whether same bacteria were found with the two methods.

**Figure 5:**
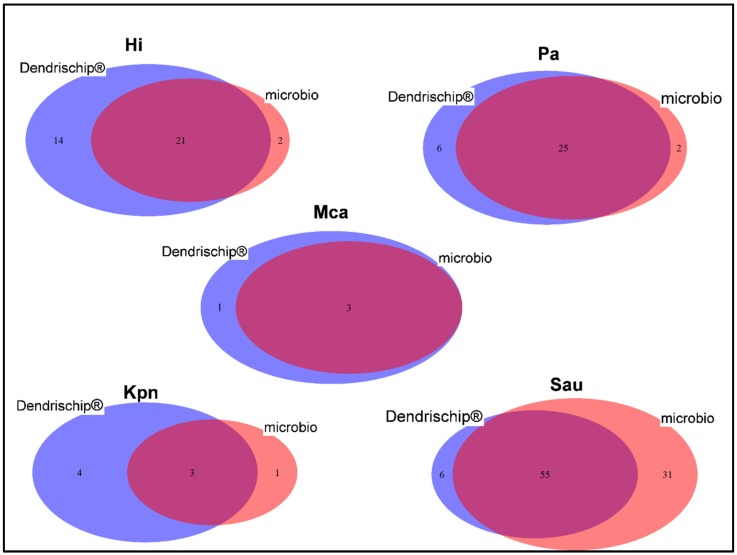
Venn diagram representation for the comparison of positive identification results for five pathogens between the DendrisChips^®^ technology and the conventional microbiology culture (microbio). In total, 238 samples consisting of 43 BAL, 43 sputums, 66 throat swabs, 33 naso-pharyngeal swabs, 15 ear swabs, 5 tongue swabs, 1 eye swab, 5 mouth swabs, 20 wound swabs, 6 bronchial aspirates and 1 tracheal aspirate have been used. Hi, *Hemophillus influenza*; Pa, *Pseudomonas aeruginosa*; Kpn, *Klebsiella pneumoniae*; Mca, *Moraxella catharralis*; Sau, *Staphylococcus aureus*.

To this end, we assessed the performance of our DendrisChips^®^ technology using a straightforward method that consists to apply a sensitivity criterion which is defined as the ratio between the number of times a given pathogen has been detected over the total number of samples in which this pathogen was given to be present (i.e., detected by both DendrisChip^®^ and microbiological method). Conversely, one can also apply another criterion termed “discrepancy value” that is defined as the number of times DendrisChip^®^ did not detect a given pathogen that has been actually identified by the microbiological method. This comparative analysis is illustrated by Venn diagrams in Figure 5. Results showed a better sensitivity criterion of DendriChip^®^ than culture method for *Haemophilus influenzae* (95% vs. 62%) and for *Pseudomonas aeruginosa* (94% vs. 82%). An apparent higher performance for *Klebsiella pneumoniae* and *Moraxella catharralis* was also obtained with our technology but these values must be taken with caution because of the low number of clinical samples containing these pathogens. On the other hand, the sensitivity criterion to detect *Staphylcoccus aureus* was apparently lower with our technology than with the microbiological culture, giving rise to a “discrepancy rate” of 54%. Due to this unexpected high discrepancy, we re-evaluated the microbiology result using a specific PCR assay for this pathogen developed by BD diagnostic [41]. We found that 21 out of the 31 samples positively identified as *Staphylococcus aureus* by the microbiological culture were not confirmed by the BDmax PCR assay, whereas those identified by DendrisChip^®^ were confirmed by this PCR assay (data not shown). Therefore, one can conclude that, even with this latter pathogen, our technology was as correct as the culture method. Finally, *Bordetella pertussis*, *Legionella pneumophila*, *Neisseira meningitidis*, *Chlamydiae pneumoniae* and *Mycoplasma pneumoniae* were never identified by either of the two methods in the 238 clinical specimens. Their absence was not due to either technical or cultivation mistake, since, by spiking these bacteria in clinical extracted DNA, we were able to detect them with our DendrisChip^®^ technology.

## 4. Discussion

The ability to detect in a same sample all potential causative agents associated with an infectious disease is going to institute a paradigm shift in the in vitro diagnostic that should revolutionize the field of clinical microbiology [5,6]. In this regard, we report here on the development and validation of a DendrisChip^®^ technique able to detect and discriminate in a single test eleven pathogen bacteria that are reported to be associated with the respiratory tract infection, a disease registered as the first of the leading causes of death in high risk patients [41,42]. We succeeded in this endeavor by raising two major locks which characterize the technology of DNA microarrays. The first limitation lies in the sensitivity of detection which in part depends on the surface chemistry to immobilize the oligoprobes and get them highly accessible to their DNA/RNA targets. To this end, DendrisChips^®^ exploited our previous works showing that the functionalization of glass surface with spherical neutral phosphorus dendrimers [43] results in 10–100 times higher accessibility of DNA/RNA targets to the probes than with other surface chemistry [28]. This greater accessibility expressed as signal/noise ratio could therefore explain our ability to assign specific and selective capture probes for each of the eleven pathogens since DNA targets from exclusion bacteria (bacteria of the same genus but of different species) do not cross-hybridize with probes designed for the inclusion bacteria. This excellent probe accessibility could also account for the good sensitivity of our DendrisChips^®^, which allows reaching a limit of detection (LOD) in the range of 10^4^ cell per mL. Of note, the LOD was found to be one log lower when the sensitivity test was carried out with serial DNA dilution, rather than with DNA extracted from serial dilution of the bacterial culture. This difference can be due to DNA kit extraction per se for which it is recommended to work with 10^6^–10^7^ cells/mL for optimal yield of DNA extraction. Nevertheless, the sensitivity of our DendrisChips^®^ technology agrees with the cutoff value of ≥10^5^ CFU/mL for considering any pathogen bacteria as clinically significant [23,44].

The second limitation of traditional DNA microarray resides in the ability to readily decode pixels values from images analysis into practical biological information. Here, the originality of our microarray-based multiplexing diagnostic was to exploit statistical learning methods to deliver a decision-making support for the biologist/clinician that is expressed as a probability of presence/absence of bacteria in clinical samples. As the decision algorithm is based on a training database of hybridization profiles of bacterial samples, the accuracy of the decision-making support shall dramatically increase with the enrichment of this training databases, which is the intrinsic property of using the Random Forests model [32] for this purpose. In addition, the attribution of specific probe patterns for each of the pathogens can be obtained using this machine learning method. Therefore, our DendrisChips^®^ technology is highly evolvable as any additional bacterial could be searched for, provided capture probes have been designed for these new species/strains and the diagnostic algorithm has been updated accordingly. In conclusion, our DendrisChips^®^ technology has the power to provide a diagnostic in less than 4 h after DNA extraction, thus accelerating the medical decision making with regards to respiratory tract infectious disease, and by extension, this technology can be applied to other infectious diseases as well as to other application fields in which bacterial control is required such as in nutrition and food quality.

However, we have to acknowledge that our technology is still at the stage of a pre-prototype which presents several limitations. A first technical concern is that, unlike the multiplex real-time PCR and RT-PCR assays, our technology is not quantitative yet. In fact, DNA microarrays have been mainly developed as an exploratory tool to investigate global genomic variabilities or transcriptomic profiling between different species or different biological situations for the same/different organisms [45,46,47]. Quantification of the hybridization signals shall be in theory possible but it will inevitably encounter two main difficulties. On the one hand, the quantification signal has to be determined for each probe–target couple and on the other hand, these values will be impacted by the “matrix effect”. The latter effect actually exemplifies a major hurdle in multiplex molecular technology, as it is well established that PCR amplification of DNA fragments can be dramatically hampered due to inhibitory entities present in biological extracts. In our technology, we have circumvented this problem by adding a known amount of a synthetic DNA fragment in the biological extract prior to PCR amplification and in case of uncertainty for presence/absence of a given pathogen, DNA from this purposed pathogen can be spiked in the samples before PCR step.

A second restriction of our technology is that we have intentionally targeted our syndrome-based diagnostic to pathogen bacteria potentially implicated in respiratory diseases since most of the multiplex molecular technologies in development or already available in clinical microbiology are designed to identify virus associated with infectious diseases. The reason invoked for this strategy is to avoid misuse of antibiotic therapy. However, almost half of the respiratory tract infections that require hospitalization are caused by co-infection of virus and bacteria [2]. It is therefore relevant to identify bacterial pathogens to propose the most adequate therapeutic solution. Beside these strategic considerations, it is technically easier to develop a multiplex detection of bacteria since they all bear variable regions of rDNA in their genomes and probes designed on these regions have been shown to be sufficient for their identification [30,48,49,50]. Hence, only a couple of PCR primers are needed to amplify this nucleic acid region, which simplifies the setting of multiplex PCR conditions. Moreover, the use of our machine learning-based algorithm allowed solving the complexity from probe–target hybridization as it can attribute to each bacterium a specific probe pattern for their identification.

At variance to most of the multiplex PCR technology, our Dendrischip^®^ technology is an open system, which can raise some problems with respect to cross-contamination. However, the advantage of being an open system is that the biological sample is not lost in the process and thus it can be analyzed again by the clinician at will with complementary molecular or microbiological methods. In addition, our technology can be easily implemented in any clinical laboratories with a minimum of investment and using the panel of apparatus, such as incubators and PCR machines, that is already available in most public or private medical structures. Finally, while our technology is still at the prototype level and requires some automation, notably for liquid handling and hybridization, it has the potential to surpass the actual multiplex end-point (RT) PCR technique mainly due to its higher throughput capacity to detect a higher number of microorganisms per biological sample and to treat a much higher number of samples per day.

## 5. Conclusions

To fulfill the emergence of the syndromic approach in molecular diagnosis, we report here a rapid, robust and easy to use DNA chips based technology that allows identifying in a single sample 11 potential bacterial pathogens implicated in respiratory infectious disease. The added-value of our technology resides in the use of phosphorus dendrimers as the surface chemistry to immobilize the probes on the chips, which increases the sensitivity of detection, and in a decision algorithm based on machine learning methods that allows discriminating the bacteria in clinical samples. Overall, a diagnostic result can be obtained in less than 4 h starting from extracted DNA sample, and the sensitivity and specificity of our DendrisChip^®^ technology proved to be at least equal to microbiological cultures.

## Figures and Tables

**Table 1:** Pathogen bacterial strains used to evaluate the specificity of the designed probes from hypervariable rDNA regions in their genomes.

Bacterial Species	Name of Strain	Source
*Bordetella pertussis*	CRBIP4.10	CRBIP *
*Chlamydiae pneumoniae*	VR-1360D	ATCC *
*Haemophilus influenzae*	DEN1 ^$^	Clinical isolate *
*Klebsiella pneumoniae*	DEN2	Clinical isolate *
*Legionella pneumophila*	ATCC 33152	ATCC *
*Moraxella catharralis*	DEN3	Clinical isolate *
*Mycoplasma pneumoniae*	ATCC 29342D	ATCC *
*Neisseria meningitidis*	CIP 73.10	CRBIP *
*Pseudomonas aeruginosa*	DEN4	Clinical isolate *
*Staphylococcus aureus*	ATCC 700699DQ	ATCC *
*Streptococcus pneumoniae*	DEN4	Clinical isolate *

* Bacterial samples were provided by Bio Pôle and IFB (Institut Fédératif de Biologie de Toulouse). All bacteria strains used in this panel were sequenced on the 16S rDNA region of interest to confirm their identification.

**Table 2:** Source of exclusion bacterial strains used to assess the oligo probes specificity.

Bacterial Strains/Species	Source *
*Bordetella bronchiseptica*	Clinical isolate
*Haemophilus parainfluenzae*	Clinical isolate
*Haemophilus aphrophilus*	Clinical isolate
*Haemophilus haemolyticus*	Clinical isolate
*Klebsiella oxytoca*	Clinical isolate
*Legionella anisa*	Clinical isolate
*Legionella spp*	Clinical isolate
*Moraxella osloensis*	Clinical isolate
*Pseudomonas spp*	Clinical isolate
*Staphylococcus epidermidis*	Clinical isolate
*Staphylococcus hominis*	Clinical isolate
*Staphylococcus haemolyticus*	Clinical isolate
*Staphylococcus schleiferi*	Clinical isolate
*Streptococcus parasanguinis*	Clinical isolate
*Streptococcus pseudopneumoniae*	Clinical isolate
*Streptococcus mitis*	Clinical isolate
*Streptococcus oralis*	Clinical isolate

* Clinical isolated provided by Bio Pôle and IFB (Institut Fédératif de Biologie de Toulouse). Identification of the species was verified by sequencing of the 16r DNA.

**Table 3:** Evaluation of the limit of detection (LOD) of the method and comparison with real-time PCR.

Bacteria	LOD (CFU/mL) *	
	DendrisChips^®^	Real-Time PCR
*Haemophilus influenzae*	10^3^	10^2^
*Klebsiella pneumoniae*	10^3^	10^2^
*Pseudomonas aeruginosa*	10^3^	10^3^
*Streptococcus pneumoniae*	10^4^	10^4^
*Moraxella catharrali*	10^3^	10^3^
*Staphylococcus aureus*	10^4^	10^4^

* Data obtained from three independent replicates.

## Supplementary Materials

The following are available online at http://www.mdpi.com/2075-4418/8/4/77/s1.
