# Supplementary material for: Innovative DendrisChips® Technology for a Syndromic Approach of In Vitro Diagnosis: Application to the Respiratory Infectious Diseases
Source: Diagnostics (Basel). 2018 Nov 11;8(4):77. doi: 10.3390/diagnostics8040077 (PMC6316573; doi:10.3390/diagnostics8040077)
Supplement: Supplementary file 1 [file diagnostics-08-00077-s001.zip › Senescau_FigSupp.pptx]

## Slide 1
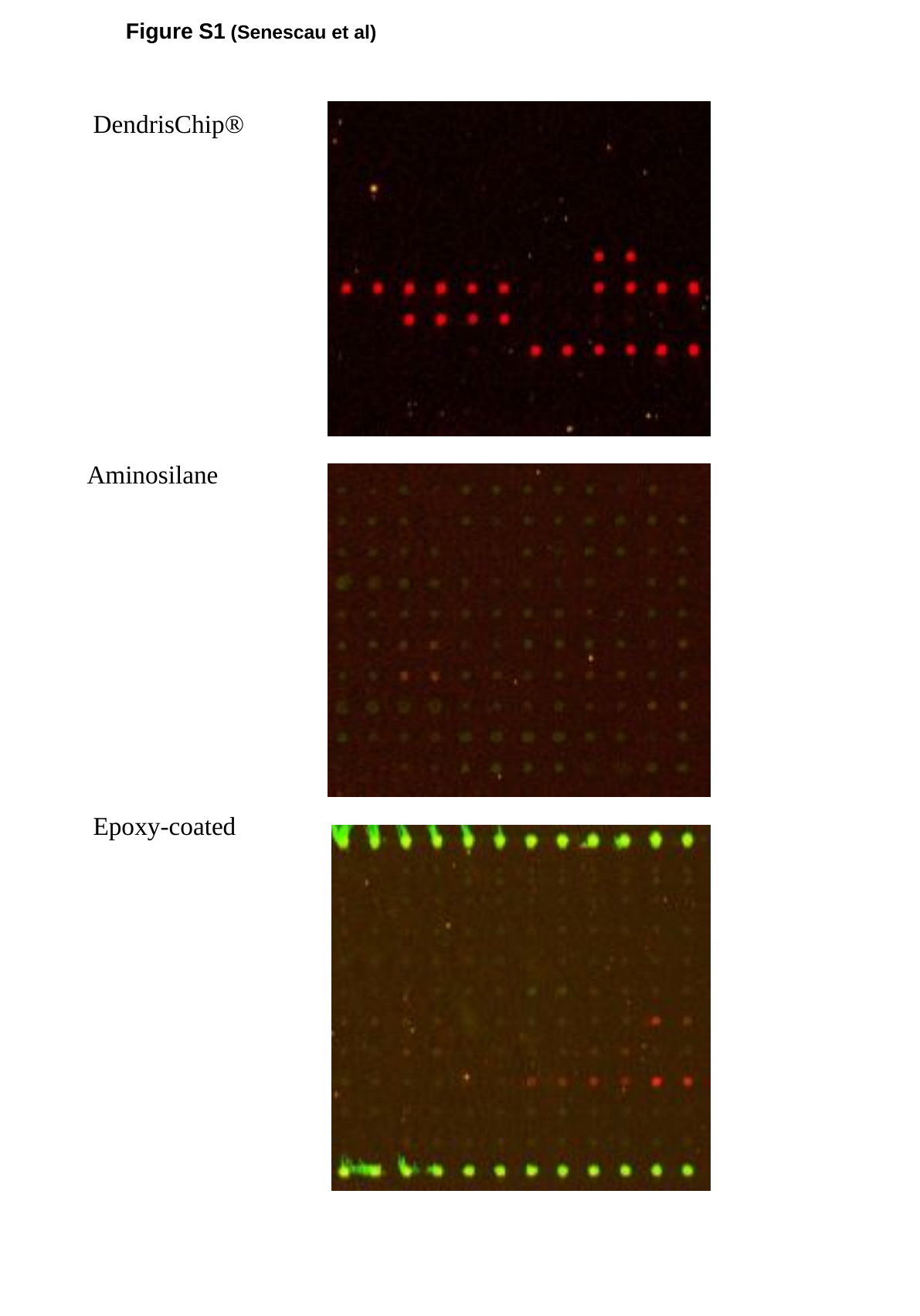

Figure S1 (Senescau et al)
 DendrisChip®
Aminosilane
 Epoxy-coated

## Slide 2
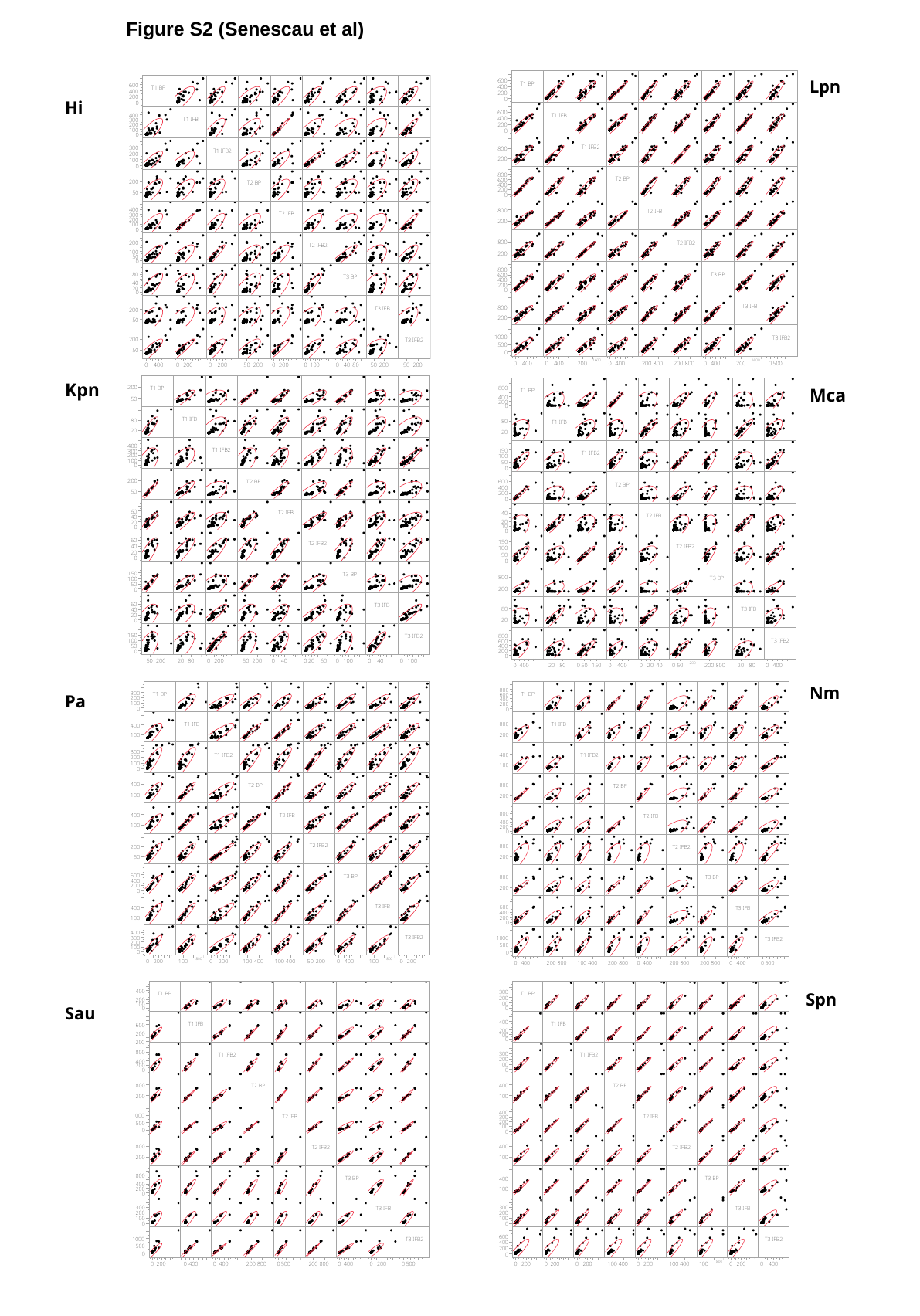

Figure S2 (Senescau et al)
Lpn
Hi
Kpn
Mca
Nm
Pa
Spn
Sau

## Slide 3
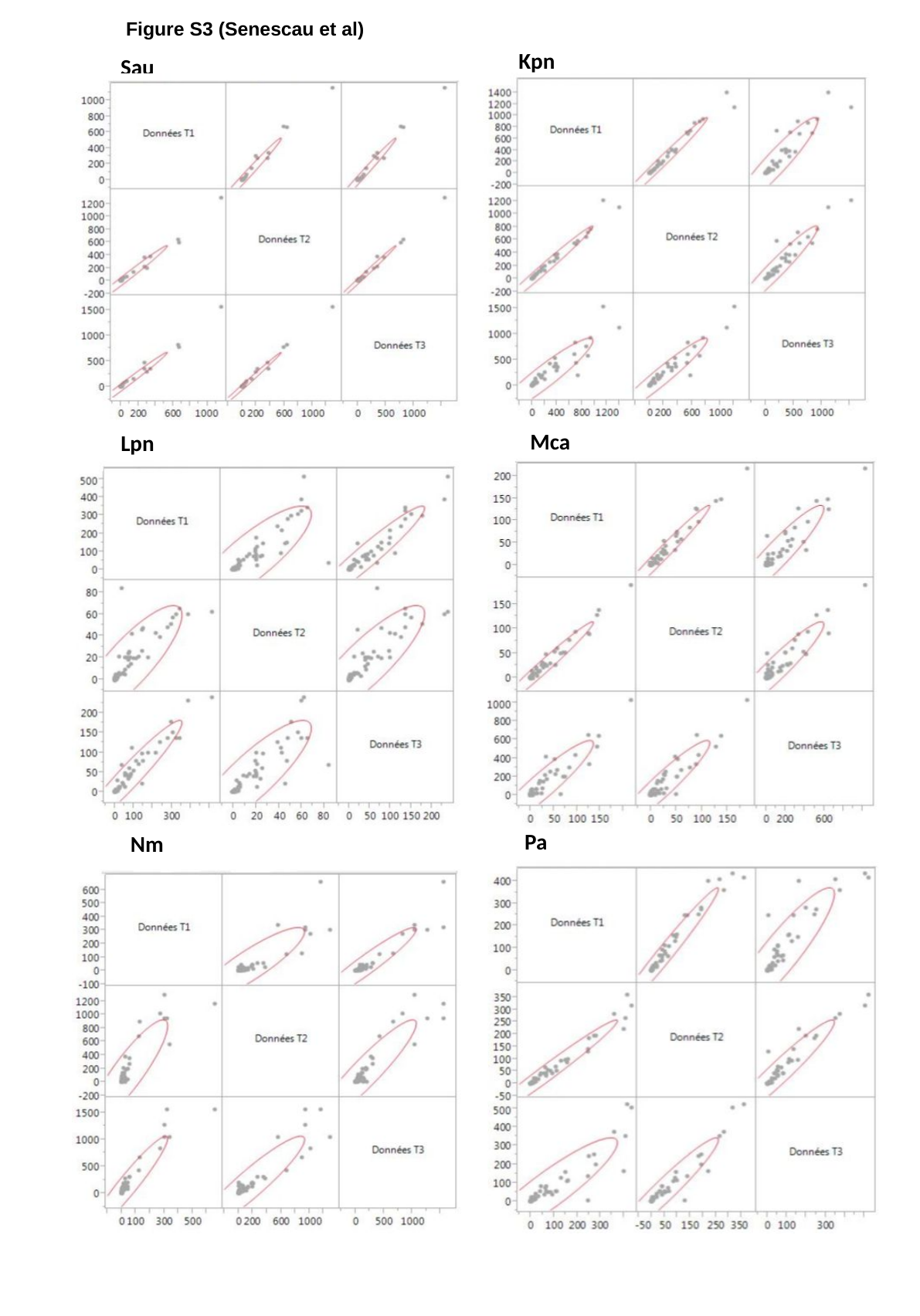

Figure S3 (Senescau et al)
Kpn
Sau
Mca
Lpn
Pa
Nm
